# The Davos Alzheimer’s Collaborative Healthcare System Preparedness US Early Detection of Cognitive Impairment Program in primary care: Methodology

**DOI:** 10.1186/s12875-026-03312-7

**Published:** 2026-04-28

**Authors:** Michael Hornbecker, Monica Zigman Suchsland, Tim MacLeod, Mary Jo Koppenhofer, Katherine J. Selzler, Phyllis Ferrell, Amy Deckert

**Affiliations:** Davos Alzheimer’s Collaborative, Wayne, PA USA

**Keywords:** Early detection, Primary care, Implementation science, Program evaluation, Methodology, Cognitive assessment

## Abstract

**Background:**

Alzheimer’s disease and related disorders (ADRD) pose a growing global health challenge, with cases expected to nearly triple by 2050. Early detection is critical for improving outcomes, yet most cases remain undiagnosed. Integrating routine cognitive assessments into primary care can provide timely access to treatments, and improve disease management and patient outcomes. The Davos Alzheimer’s Collaborative Healthcare System Preparedness (DAC-SP) program created an Early Detection Blueprint (dacblueprint.org), a digital, open-access microsite which translates evidence-based implementation strategies and resources into a practical operational guide for healthcare systems to establish early detection programs in primary care.

**Methods:**

To help bridge the gap between research and real-world practice, DAC-SP launched the US Early Detection of Cognitive Impairment Program in September 2024. This program supports 10 diverse healthcare systems across the United States through grant funding and a core curriculum including self-directed, peer and expert learning to design, implement, and sustain routine cognitive assessment programs in primary care settings. Informed by the Consolidated Framework for Implementation Research (CFIR), program evaluation data will be collected via surveys, monthly implementation reports, and semi-structured qualitative interviews. Using a concurrent mixed-methods program evaluation, this study will assess the implementation outcomes of adoption, implementation, and sustainability of the routine cognitive assessment programs across sites.

**Results:**

The US Early Detection Program is expected to conclude in July of 2026, with sustainability assessments completed by December 2026 and results by March 2027. Findings will include cross-site changes in the number of routine cognitive assessments completed over time and changes in healthcare provider attitudes towards early detection and implementation of adapted clinical workflows. Additional learnings from the US Early Detection Program will include barriers and facilitators to the early detection of cognitive impairment in primary care, and site-specific factors that influenced adoption of routine cognitive assessments across diverse practice settings. Cross-site learnings and feedback will inform revisions to a US-specific version of the Early Detection Blueprint for future US healthcare systems.

**Conclusion:**

The DAC-SP US Early Detection Program aims to modernize detection, diagnosis, and care of Alzheimer’s disease and related disorders by expanding the routine use of cognitive assessments in primary care and generating actionable insights to drive improvements across the US healthcare landscape.

**Supplementary Information:**

The online version contains supplementary material available at 10.1186/s12875-026-03312-7.

## Background

Alzheimer’s disease and related disorders (ADRD) are among the most pressing global health challenges today. With an aging global population, the prevalence of dementia is expected to rise from 57.4 million in 2019 to 152.8 million by 2050 [1]. Healthcare systems are unprepared to address the growing number of individuals with dementia and cognitive impairment, with cases remaining largely undetected or detected late in the disease progression. In the United States, common barriers to the early detection of cognitive impairment include a lack of clinical guidelines and provider training for cognitive assessments, primary care capacity constraints, limited reimbursement, and stigma of the disease [2, 3], highlighting the critical need for scalable and effective solutions to improve early detection.

There is reason for optimism in the detection and management of ADRD with emerging clinical innovations and novel therapeutics that promise to revolutionize care for patients with Alzheimer’s disease. The increasing prevalence of ADRD and emerging innovations for early detection underscores the critical need for effective strategies to address the gap in healthcare system readiness [4].

Early detection of cognitive impairment enables providers to evaluate patients for potentially reversible causes, provides an opportunity to optimize management of comorbid conditions, and allows providers and patients to have impactful conversations regarding lifestyle modifications and legal, financial, and end of life matters [3, 5]. Moreover, early detection facilitates timely access to appropriate clinical trials and therapeutic interventions, including disease-modifying treatments that are most effective in those with mild cognitive impairment or early Alzheimer’s disease [6, 7]. An early ADRD diagnosis enables access to dementia care programs such as CMS GUIDE, modeled after other comprehensive programs that demonstrated improved care outcomes and lower costs [8, 9]. The longitudinal nature of the relationship between patients and their primary care provider uniquely positions primary care to play a crucial role in early detection.

To overcome strained primary care capacity and limited health system engagement in early detection, the Milken Institute Future of Aging recommends the integration of cognitive assessment tools into clinical pathways efficiently and in a way that minimizes burden on already overworked primary care clinicians [10]. The Davos Alzheimer’s Collaborative System Preparedness (DAC-SP) program aims to make this recommendation a reality, leveraging implementation science methods to increase rates of cognitive screening, early detection, and accurate diagnosis of Alzheimer’s disease in health systems worldwide [4]. The Davos Alzheimer’s Collaborative (DAC) is a pioneering global initiative pursuing the goal to accelerate innovation and deliver solutions around the globe to transform Alzheimer’s disease research, prevention, and care.

In 2021, DAC-SP launched the Early Detection Flagship Program, the first multi-site implementation initiative, in seven sites across six countries (Brazil, Jamaica, Japan, Mexico, Scotland, and two sites in the US). The goal of this program was to understand the barriers and facilitators to implementing digital cognitive assessments (DCA) and a blood biomarker (BBM) test in existing primary and non-specialty care pathways [11, 12]. Practical learnings from this program were synthesized into the Early Detection Blueprint microsite (Blueprint; www.dacblueprint.org), an open-access resource of best practices for optimizing Alzheimer’s disease detection, diagnosis, and care. The Blueprint translates evidence-based implementation strategies and resources into a practical, comprehensive, implementation guide. The Blueprint was designed to be broadly applicable across settings (e.g., model, payer, size), for use by healthcare systems worldwide to assist in the development of early detection programs in primary care.

The information architecture and user flows for the Blueprint microsite were co-designed with multinational Early Detection flagship site leaders and the content was further evolved based on results from the qualitative research arm of the flagship program [13]. The microsite design is intended to optimize user experience, with digital design and development executed by technology partner Sunnyside Studio.

Findings from the multi-site Early Detection Flagship Program suggest that it was both feasible and acceptable to incorporate early detection tools into primary care [12]. The implementation evaluation revealed significant barriers to systematic set-up and delivery of early detection programs in U.S. healthcare systems, including a lack of practical guidance regarding the set-up and delivery of these programs and a lack of primary care leaders to serve as program “champions” [13]. To address these barriers, DAC-SP launched the US Early Detection Program in September 2024 to support 10 diverse US healthcare systems in designing, implementing, and sustaining routine cognitive assessment programs in primary care. The primary goal of the program is to increase the number of routine cognitive assessments completed in primary care settings. This paper describes the methodological approach for the US Early Detection Program and findings will be published in subsequent publications.

## Methods

### Design and objectives

This study uses a concurrent mixed methods design to evaluate the multi-site, pragmatic US Early Detection Program, incorporating approaches from implementation science [14, 15] and theory-based system and individual behavior change [16–18]. The mixed methods evaluation will be guided by the Consolidated Framework for Implementation Research (CFIR [19]). DAC-SP will support sites by providing grant funding and core curriculum components, with self-directed, peer, and expert learning to guide their development and implementation of routine cognitive assessment programs in primary care. Site implementation teams will independently develop the necessary protocols, change management processes, tools, training and resources for adoption and sustainment of their routine cognitive assessment programs. DAC-SP will conduct a cross-site program evaluation to assess site characteristics that guided development of their routine cognitive assessment program and evaluate the barriers and facilitators that influenced actual adoption, implementation, and sustainability in the primary care setting. Site feedback and implementation progress reporting throughout the program will inform iterative, US-specific refinements to the Early Detection Blueprint.

### Participants

DAC promoted an open call for applications to the US Early Detection Program using social media and email distribution by DAC and other non-profit organizations with relevant networks and reach. Ten sites were selected following evaluation of submitted proposals by an independent panel of reviewers, assessing capabilities to implement cognitive assessments in primary care, and the potential reach, scale, and sustainability of proposals. Prioritization of sites further emphasized a cross-site diversity across geographic location (national, West, Midwest, Southeast, Northeast), healthcare system type (national community, small community, large regional, academic), payer mix, setting (urban, suburban, rural), and patient population (age, gender, race, ethnicity, social determinants of health).

Selected site applicants identified implementation teams, including operations and clinical leaders from primary and specialty care clinics within their healthcare system to design and implement a routine cognitive assessment program in primary care. Site implementation teams will identify clinics or sets of healthcare providers (HCPs) to participate in implementing their site-specific routine cognitive assessment program. Providers that are trained on the program or participate in the implementation process will be eligible to complete the HCP confidence and attitudes survey (Appendix 1).

Site implementation teams will set their own cognitive screening eligibility criteria. Patients meeting site-defined requirements will be considered eligible for routine cognitive assessment and will be tracked as part of the monthly reporting of aggregated clinical cognitive assessment data.

### Program design and delivery

#### Self-directed learning

DAC-SP developed a core curriculum based on resources in the Early Detection Blueprint to provide implementation guidance for sites on stakeholder engagement, clinical workflow adaptation, provider training, billing, and outcomes evaluation (Table 1). The implementation guidance and resources are intended to facilitate site-specific selection of cognitive assessment tools, creation of clinical workflows, and the training and education of HCPs within each site. Sites are required to complete an assignment monthly as part of their program development and evaluation.

**Table 1:** US Early Detection Program Curriculum

Session		Curriculum Topics
Plan Module	1	• Evaluate healthcare system context and readiness • Create a program proposal • Determine core stakeholder team and change management approach
	2	• Select your cognitive assessment and define rationale
	3	• Define your evaluation framework, including program metrics and monitoring plan • Assemble your project team, including organizational chart and communication timeline
	4	• Develop protocols and create clinical workflow chart • Develop education and training materials, including training plan for clinicians and protocol for disclosing cognitive assessment results
	5	• Barriers and mitigation strategies
Implement Module	6	• Develop comprehensive project plan with milestones • Define approaches for identifying and selecting appropriate patients for cognitive assessments
	7	• Set up clinic space, EHR, and IT systems • Pilot and test clinical workflows • Reflect on challenges and improvements • Initiate program roll-out
	8	• Barriers and mitigation strategies
Monitor and Evaluate Module	9	• Monitor progress and identify opportunities for improvement • Create a dissemination plan with concrete strategies for scaling the program across your health system
	10	• Barriers and mitigation strategies

To learn more about the curriculum topics, see www.dacblueprint.org

*Abbreviations EHR*  Electronic health record, * IT*  Information technology

The Early Detection Blueprint microsite (www.dacblueprint.org) is organized using a modular approach, including sections on planning, implementation, and monitoring and evaluation, with corresponding goals, actions and resources. Brief videos will be distributed monthly to site implementation teams to support their asynchronous review of the Blueprint materials, and orienting sites to the assignment.

#### Peer and expert learning

US Early Detection Program site implementation teams will convene monthly as a community of practice (CoP) for peer-to-peer experiential learning and knowledge sharing. Collaboration at healthcare communities of practice can be effective at changing practice and improving clinical outcomes [20]. US Early Detection CoP meetings include a semi-structured agenda designed to foster discussion across site implementation teams to address common barriers, design and adapt clinical workflows, enhance billing and coding strategies, and to plan for sustainability. Additional peer learning is promoted through group meetings (*n* = 2–3 sites) to review Blueprint modules and assignments, best practices, and barriers and mitigation strategies. Throughout the US Early Detection program, DAC will encourage sites to share feedback on the Blueprint and provide suggestions for US-specific content and resources.

Expert implementation guidance is also facilitated through the DAC program by connecting sites to consultants with expertise on billing and coding and/or early detection in primary care settings.

### Program evaluation and data collection

The DAC-SP research team will collect quantitative and qualitative data to understand the site characteristics that guide routine cognitive assessment program development (Table 2) and to evaluate the barriers and facilitators that influence actual adoption, implementation, and sustainability in the primary care setting (Table 3). Additionally, findings will inform scalable revisions to the Blueprint and the US Early Detection Program delivery model for future use by healthcare systems.

**Table 2:** Data Sources and CFIR Implementation Context Constructs

Research Question	Data Sources	CFIR Domain	Determinants
What characteristics at the site guided the routine cognitive assessment program development?	• PRIUS • Midpoint site implementation team interview • Blueprint assignments and implementation surveys • Baseline HCP survey • Community of Practice	Innovation Characteristics	Evidence and rationale for the routine cognitive assessment program and compatibility of clinical workflows
		Individual Characteristics	Motivation and opportunity of HCPs to deliver the routine cognitive assessment program
		Inner Setting	Relative priority within clinics; access to training and resources
		Outer Setting	Partnerships, system-wide support, and cost-benefit rationale
		Implementation Process	Strategies used to implement the routine cognitive assessment program

**Table 3:** Data Sources and CFIR Outcomes

Research Question	Data Sources	Implementation Outcome	Measures
What barriers and facilitators influenced actual adoption, implementation, and sustainability in the primary care setting?	• Monthly clinical data • Baseline and post- program HCP surveys • Post-program and sustainability Implementation team interviews	Adoption	Number of providers trained
			HCP-reported confidence & engagement
		Implementation	Change in cognitive assessments baseline vs. post-implementation
		Sustainability	Assessment of routine cognitive assessment program 6 months post-program

#### Clinical cognitive assessment data

Implementation teams will submit monthly reports summarizing clinical implementation activities over the program implementation timeline. Monthly data will be reported in aggregate at the site level and will include number of patients eligible for screening, number screened, cognitive assessment results (normal, abnormal, indeterminate/intermediate), the number and nature of referrals generated after the assessment, the number of clinics that implemented the program, and the number of HCPs recruited for participation.

#### Surveys

Implementation teams will be asked to complete a brief online survey during each month of the planning phase to assess the relevance, usefulness, and satisfaction with curriculum and delivery of the US Early Detection Program. The survey was developed for this program and uses a 5-point Likert scale, with two open-ended questions to prompt participant feedback. Higher scores denote greater relevance, usefulness, outcome expectations, and satisfaction. In addition, implementation teams will track implementation progress on a monthly basis using the Prospectively Reported Implementation Update and Score (PRIUS) method [21]. Using the prompt, “What are some things that happened over the past month that seem relevant from your perspective to the implementation of this project?”, relevant events reported will be assigned a score ranging from − 3 to + 3. Positive scores denote a positive impact on the implementation process (i.e., facilitators) while negative scores denote a negative impact (i.e., barriers). The numerical score denotes the magnitude of the impact. Sites will submit the PRIUS electronically to DAC.

Eligible HCPs will be asked to complete a brief online survey at baseline and post-implementation to assess: (1) individual attitudes towards the importance of early detection; (2) the ease of implementing the routine cognitive assessment program; (3) efficacy expectations (i.e., the extent to which they are confident they can execute their site-specific clinical workflow); and (4) demographics and professional experience. The survey will use a 5-point Likert scale with higher scores denoting greater perceived importance, ease of implementation, and efficacy expectations. This survey was developed for the US Early Detection Program and is available, for reference, in Appendix 1.

#### Qualitative interviews

Qualitative interviews with implementation teams will be conducted at the midpoint and end of the program. Semi-structured interview guides were developed for this study to understand implementation team perspectives on incorporating routine cognitive assessments into primary care workflows and to identify site‑ and system‑level barriers and facilitators influencing implementation of the routine cognitive assessment program. At 6-months post-program, a follow-up interview with site implementation teams will explore routine cognitive assessment program sustainability across sites.

At program completion, a subset of HCPs will be purposively sampled to participate in qualitative interviews. The semi-structured interview guide for HCP participants was developed for the US Early Detection Program to help us further understand HCP attitudes and confidence, and identify barriers and facilitators that influence adoption, implementation, and sustainability of routine cognitive assessments in their primary care setting. HCPs will be invited to participate in these interviews based on their responses to the post-implementation HCP survey (i.e., sampling for low, mid, and high scores).

Qualitative interviews will be scheduled for 30 min and conducted by a DAC-SP qualitative researcher with a second DAC-SP researcher transcribing the interview in real time. Interviews will take place either in-person or over video conference and will be recorded. Recordings will only be used to verify the accuracy of the interview transcription and will be deleted following verification.

### Planned analyses

#### Clinical cognitive assessment data

Clinical cognitive assessment data will be consolidated across sites to evaluate the adoption and implementation success of the routine cognitive assessment program. Rates of cognitive assessments and rates of clinical referrals will be summarized over the course of the program. We will estimate rate ratios comparing the start and end of the implementation period to assess changes in the number of cognitive assessments completed over time. Rates will be modeled using negative binomial regression with a log (time in months) offset, adjusting for site-level characteristics (cognitive assessment tool, implementation setting, size).

#### Survey data

Survey responses will be aggregated at the site level and across sites. Descriptive statistics (i.e., frequency, mean, SD) will be calculated to summarize responses. Pre-post program comparisons using independent t-test will be conducted to evaluate changes in HCP attitudes, motivation, and confidence over time. PRIUS scores and events will be summarized within and across-sites to evaluate program-wide progress, facilitators and barriers.

#### Qualitative interview data

Two DAC-SP research team members will debrief post-interview (i.e., within 48 h) to review reflections, interview notes, and transcription. The coding approach will be guided by conventional content analysis [22]. A coding template with program-specific CFIR coding framework will be developed to facilitate rapid deductive coding of interview transcripts [23], which will be followed by inductive coding to capture emergent themes. Two DAC-SP research team members will meet to review where codes converge and diverge, and the patterns that reflect barriers and facilitators to implementation. Codes will be clustered into higher order groups and emergent themes. Themes will be synthesized and aligned with the evaluation aims and implementation constructs.

#### Mixed methods analyses

Survey results and clinical cognitive assessment results will be triangulated with qualitative findings to examine the influence of site characteristics, barriers, and facilitators on routine cognitive assessment adoption, implementation, and sustainability. Triangulation across quantitative and qualitative data sources will be guided by the Consolidated Framework for Implementation Research (CFIR) [19]. Tables 2 and 3 outline the triangulation approach used for the research questions across data sources, CFIR constructs, outcomes and measures.

## Discussion

Early detection of cognitive impairment in primary care is pivotal in ensuring timely access to appropriate care and interventions, including opportunities for patients to reduce risk through lifestyle modifications and improved chronic disease management, and to benefit from case management and interventions that require an ADRD diagnosis to determine eligibility (e.g., CMS GUIDE Model, disease-modifying treatments, and/or appropriate clinical trials). The successful implementation of early detection programs in primary care settings depends upon the integration of validated cognitive assessments into clinical workflows in a manner that minimizes burden on primary care clinicians, with clear guidelines for appropriate evaluation and management [10]. The DAC-SP US Early Detection Program addresses this important need for practical guidance on standardizing early detection of cognitive impairment in the United States. Moreover, the program offers the opportunity to convene healthcare leaders, often siloed within their own institutions, within a learning platform intentionally designed to foster collaboration and share expertise around common barriers to early detection.

We identified a diverse sample of healthcare organizations to participate in the US Early Detection Program to mitigate the risk for bias and limited generalizability of the findings. Through the implementation evaluation, we aim to identify a comprehensive resource of implementation guidance that can be applied by other US healthcare organizations to support tailoring of routine cognitive assessment programs for their setting. The variability of approaches to program design and implementation across sites will allow us to generate learnings that will be broadly applicable, regardless of the cognitive assessment tool, staffing model, billing strategy, or clinical workflow implemented.

## Conclusion

Through grant funding, Blueprint-based curriculum, and peer and expert learning, the DAC-SP US Early Detection Program aims to increase dissemination and improve the rates of early detection for cognitive impairment across US primary care settings. This program is expected to conclude in July 2026, with sustainability assessments completed by December 2026 and results anticipated in March 2027. This program evaluation will contribute practical guidance on tailoring program design and tactics to support implementation of routine cognitive assessment programs across diverse US healthcare organizations. Learnings from this program will be incorporated into the DAC-SP Blueprint as implementation guidance for other healthcare systems.

## Supplementary Information

Supplementary Material 1.

Supplementary Material 2.

## Data Availability

The implementation survey distributed to participating healthcare providers is included as Appendix 1 (Supplementary materials). This paper reports on the methodology and no data are available. Additional program delivery and evaluation materials (e.g., qualitative interview guides, monthly feedback surveys) will be made available by the corresponding author, AD, upon reasonable request.

## Abbreviations

ADRD: Alzheimer’s disease and related disorders
BBM: Blood biomarker
CFIR: Consolidated Framework for Implementation Research
CMS: Centers for Medicare & Medicaid Services
CoP: Community of practice
DAC-SP: Davos Alzheimer’s Collaborative Healthcare System Preparedness
DCA: Digital cognitive assessment
GUIDE: Guiding an Improved Dementia Experience
HCP: Healthcare provider
PRIUS: Prospectively Reported Implementation Update and Score
