# Supplementary material for: The Davos Alzheimer’s Collaborative Healthcare System Preparedness US Early Detection of Cognitive Impairment Program in primary care: Methodology
Source: BMC Prim Care. 2026 Apr 28;27:234. doi: 10.1186/s12875-026-03312-7 (PMC13274047; doi:10.1186/s12875-026-03312-7)
Supplement: Supplementary file 1 — Supplementary Material 1. [file 12875_2026_3312_MOESM1_ESM.docx]

**Appendix 1**

**DAC-SP US Early Detection Program Healthcare Provider Implementation Survey**

You are being invited to complete this survey based on your training on using a brief cognitive assessment tool for the early detection of cognitive impairment program at [*Site]*

4-digit unique identifier _____

**About You and Your Practice**

Please respond to the following questions honestly and accurately. Your responses will not be linked to your name.

1. **What is your profession? (select all that apply)**

Physician

Nurse practitioner

Physician assistant

Psychologist

Nurse

Administrator

Other ___ (please specify)

1. **How long have you been in practice? (include years of residency if applicable)**

___ years

1. **What is your medical specialty? (Select all that apply).**

Family medicine

Internal medicine

Geriatric medicine

Neurology

Psychiatry

Other ____ (please specify)

1. **Approximately what percentage of patients in your practice have cognitive impairment or dementia?**

_____ %

1. **Approximately what percentage of patients in your practice are over 65 years of age?**

_____ %

1. **How many years have you worked at your health system?**

**____** years

1. **What is your age**?

___ years

1. **What is your gender**?

Male/female/other

1. **What is your race? (Select all that apply)**
   White
   Black or African American

Hispanic or Latino
American Indian or Alaska Native
Asian
Native Hawaiian or Pacific Islander

Middle Eastern or North African
Other please specify):__________________

**Your Perceptions on Early Detection of Cognitive Impairment**

The following questions ask about your perspective on incorporating a brief cognitive assessment tool into your clinical practice. The survey takes about 5-10 minutes to complete.

Please use the scale below to indicate the extent to which you agree or disagree with the following statements. If the question is not relevant to you, please select "N/A".

Response options:

- Strongly disagree, disagree, neither agree nor disagree, agree, strongly agree

N/A

| 1 | A brief cognitive assessment tool helps me evaluate my patients with concerns about their memory and thinking |
| --- | --- |
| 2 | I trust that a brief cognitive assessment tool will accurately detect cognitive impairment in my patients |
| 3 | I am more confident making a diagnosis of mild cognitive impairment when using a brief cognitive assessment tool in addition to my clinical judgment |
| 4 | Many of my patients over 65 ask me to test their memory and thinking |
| 5 | Many of my patients over 65 tell me that they are concerned about their memory and thinking |
| 6 | It is easy for me to administer a brief cognitive assessment tool in my clinic |
| 7 | It is easy for me to document cognitive assessment results in the patient’s medical record |
| 8 | It is easy for me to access the results of a brief cognitive assessment tool in my clinic |
| 9 | It is easy to administer a brief cognitive assessment tool as part of the Medicare wellness visit in my practice |
| 10 | Early detection of dementia can help patients and their caregivers plan for the future |
| 11 | It is important to discuss brain health with my patients |
| 12 | A brief cognitive assessment tool encourages me to discuss brain health with my patients, regardless of the test result |
| 13 | I am confident I can discuss the benefits and limitations of a brief cognitive assessment tool with my patients |
| 14 | I am confident I can discuss the benefits and limitations of a brief cognitive assessment tool with my patients, even if I am pressed for time |
| 15 | I am confident that I know how to administer a brief cognitive assessment tool |
| 16 | I am confident I can explain the results of a brief cognitive assessment tool to my patients |
| 17 | I am confident I can explain the results of a brief cognitive assessment tool to my patients, even if I am pressed for time |
| 18 | I am confident I know the appropriate clinical workup for my patients after their cognitive assessment |
| 19 | I am confident I know how to utilize new clinical tools (such as EHR order sets) that were created to support my cognitive workup |
| 20 | I am confident that I can code appropriately for billing the diagnostic workup for cognitive impairment (e.g., labs, MoCA, imaging) |
| 21 | It is appropriate to administer a brief cognitive assessment tool to all patients over 65 |
| 22 | I am optimistic about the recent advances in the diagnosis and treatment of Alzheimer’s disease |
| 23 | An early diagnosis of cognitive impairment is important for patient referrals to time-sensitive interventions |
| 24 | From a financial perspective, it is worthwhile for me to administer brief cognitive assessment tools in my practice |
| 25 | In the next year, I plan to administer brief cognitive assessment tools to most of my patients over 65 |

26. Is there anything you would like to add? ________
