# Supplementary material for: The Davos Alzheimer’s Collaborative Healthcare System Preparedness US Early Detection of Cognitive Impairment Program in primary care: Methodology
Source: BMC Prim Care. 2026 Apr 28;27:234. doi: 10.1186/s12875-026-03312-7 (PMC13274047; doi:10.1186/s12875-026-03312-7)
Supplement: Supplementary file 2 — Supplementary Material 2. [file 12875_2026_3312_MOESM2_ESM.docx]

**Appendix 2. Key Learnings and Barriers from Previous DAC-SP Programs Informing the US Early Detection Program**

| **Domain** | **Key Learning / Barrier** | **Implication for US Early Detection Program** |
| --- | --- | --- |
| Implementation Guidance | Lack of practical, step-by-step guidance for setting up early detection programs in primary care | Development of the DAC-SP Early Detection Blueprint microsite as a structured, modular implementation resource |
| Leadership & Champions | Limited primary care leaders with time or appropriate expertise equipped to champion early detection programs | Program prioritizes identification and support of site implementation leaders ("champions") at each site |
| Workflow Integration | Difficulty embedding cognitive assessments into existing clinical workflows without adding burden | Blueprint provides guidance for developing workflows for processes and staffing models, with example training materials for clinicians |
| Stakeholder Engagement | Limited engagement of key institutional stakeholders (e.g., IT, administration, billing) early in the process | Program curriculum module 1 focuses on stakeholder mapping and change management |
| Reimbursement & Resources | Uncertainty around reimbursement and resource allocation for cognitive screening | Blueprint includes resources on billing codes and reimbursement pathways |
| Sustainability | Early detection programs often discontinued after initial enthusiasm waned | Program includes dedicated sustainability assessment and dissemination planning module |
